# Supplementary material for: Happiness at Work among Public Relations Practitioners in Spain
Source: Int J Environ Res Public Health. 2022 Mar 27;19(7):3987. doi: 10.3390/ijerph19073987 (PMC8997484; doi:10.3390/ijerph19073987)
Supplement: Supplementary file 1 [file ijerph-19-03987-s001.zip › ijerph-1627608-supplementary.pdf]

Table S1. Mean and SD obtained in the Workplace PERMA-Profiler (range from 0 to 10), by gender, age and position.

			P	E	R	M	A	Total	N	H	Total with H and N
Gender	Male	Mean	6.4727	6.7212	7.3273	6.6970	7.1394	6.8715	4.9879	6.5636	5.1333
		SD	2.05950	1.87551	1.59667	1.89679	1.56832	1.59752	2.02247	1.95302	1.43708
	Female	Mean	7.1475	7.4754	8.2186	6.8169	7.5246	7.4366	4.7322	7.2295	5.6686
		SD	1.31534	1.28416	1.25366	1.65206	1.11169	1.03312	1.95667	1.59607	0.95133
Age	Under 32	Mean	6.9624	7.2151	8.4140	6.7097	7.5699	7.3742	4.4785	7.1828	5.6536
		SD	1.84766	1.51507	1.50007	1.62683	1.18489	1.23485	2.19174	1.90166	1.15322
	32-38	Mean	6.8182	7.1333	7.6909	6.6909	7.3152	7.1297	5.0061	7.0545	5.3853
		SD	1.43561	1.60195	1.30818	1.58030	1.18707	1.21617	1.83865	1.56184	1.08998
	Over 38	Mean	7.0222	7.3667	7.6833	6.9333	7.3167	7.2644	4.9778	6.8278	5.4532
		SD	1.51187	1.48907	1.34658	1.95986	1.44767	1.33045	1.84306	1.72113	1.19528
Cargo	High management	Mean	7.4348	7.7101	7.7826	7.3986	7.7246	7.6101	4.6667	7.0145	5.7712
		SD	1.33309	1.34552	1.32817	1.52933	1.21372	1.15990	1.56031	1.67768	1.04659
	Intermediate management	Mean	6.8611	7.3403	7.9306	6.6319	7.3542	7.2236	5.1042	6.9583	5.4246
		SD	1.40218	1.39653	1.19881	1.82087	1.20559	1.16493	1.93164	1.61058	1.09486
	Senior Technician	Mean	6.7821	7.0000	7.6026	6.6538	7.2308	7.0538	4.2564	7.5000	5.5018
		SD	1.61091	1.64924	1.58330	1.66662	1.26410	1.27164	2.00955	1.51217	1.14517
	Technician	Mean	6.7758	6.9636	8.3576	6.5636	7.3758	7.2073	4.9879	7.0061	5.4364
		SD	1.77088	1.44398	1.26174	1.60615	1.11121	1.12258	2.23603	1.81952	1.11095
Total		Mean	6.9379	7.2411	7.9416	6.7797	7.4049	7.2610	4.8117	7.0226	5.5023
		SD	1.60901	1.52823	1.42612	1.72724	1.27917	1.25921	1.97515	1.73700	1.14785

Table S2. Significance coefficients of differences by gender. age and position.

	i	j	P	E	R	M	A	Total	N	H	Total with H & N	Happiness (Q16-i23)
Gender	Male	Female	0.079	0.029*	0.000*	0.895	0.236	0.069	0.507	0.040*	0.027*	0.610
Age	Under 32	32-38	0.880	0.955	0.015	0.998	0.547	0.553	0.320	0.918	0.422	0.805
		Over 38	0.991	0.848	0.011	0.903	0.371	0.803	0.380	0.388	0.510	0.939
	32-38	Over 38	0.817	0.692	0.999	0.884	0.963	0.906	0.988	0.650	0.983	0.952
Position	High management	Intermediate management	0.236	0.592	0.952	0.064	0.275	0.286	0.750	0.980	0.328	0.666
		Technician	0.144	0.049	0.129	0.069	0.483	0.318	0.844	1.000	0.426	0.331
		Senior technician	0.313	0.185	0.944	0.281	0.355	0.219	0.828	0.652	0.751	0.278
	Intermediate management	Technician	0.997	0.541	0.338	1.000	0.973	0.999	0.997	0.981	0.996	0.952
		Senior Technician	0.999	0.762	0.741	0.991	0.999	0.970	0.322	0.437	0.974	0.829
	Senior technician	Technician	1.000	0.541	0.077	0.996	0.959	0.947	0.398	0.617	0.994	0.973

* p < .05; ** p < .01; *** p < .001
